# Diagnostic accuracy of urine test strips for CSF analysis: high sensitivity in detecting blood, low sensitivity but high specificity in detecting inflammation

**DOI:** 10.3389/fneur.2026.1893760

**Published:** 2026-10-26

**Authors:** Lina Marie Albers, Franz Felix Konen, Erda Atakan, Konstantin Fritz Jendretzky, Sandra Nay, Lea Grote-Levi, Kurt-Wolfram Sühs, Thomas Skripuletz, Philipp Schwenkenbecher

**Affiliations:** Hannover Medical School, Department for Neurology, Hannover, Germany

**Keywords:** cerebrospinal fluid, erythrocytes, leukocytes, proteins, sensitivity and specificity, urinary reagent strip

## Abstract

**Background:**

Rapid cerebrospinal fluid (CSF) analysis is crucial for diagnosing and treating time-critical neurological diseases. However, thorough laboratory diagnostics are time-intensive or often unavailable in resource-limited settings. Urine test strips (UTS) may provide a simple bedside alternative, but evidence remains limited and largely restricted to single manufacturers. This study evaluated the diagnostic performance of UTS for CSF analysis and compared different manufacturers.

**Methods:**

In this prospective study, 285 CSF samples from patients with different neurological diseases were analyzed using UTS from five manufacturers. Strip results for leukocytes, erythrocytes, protein, and glucose were compared across manufacturers and against laboratory measurements. Diagnostic accuracy for detecting blood in CSF, pleocytosis, and for differentiating infectious from non-infectious conditions was assessed.

**Results:**

For erythrocyte detection, the UTS showed high sensitivity (86–95%) and negative predictive value (NPV, 92–97%). In contrast, for CSF pleocytosis, specificity and positive predictive value (PPV) were high (100%), whereas sensitivity was low (11%). UTS underestimated low leukocyte counts and showed variable performance for protein detection. For differentiating infectious from non-infectious conditions, leukocytes and glucose demonstrated high specificity and PPV. Combining parameters (≥ 2/3 positive: leukocytes, protein, glucose) yielded consistently high specificity (98%) and PPV (80–85%), but low sensitivity (13–15%) and NPV (63%). No relevant differences between manufacturers were observed.

**Conclusion:**

UTS provide a rapid, low-cost bedside tool for initial CSF assessment, with particular strength in reliably excluding blood contamination. In contrast, their utility for detecting inflammatory or infectious conditions is limited due to low sensitivity. Therefore, while UTS may support early bedside decision-making, they cannot replace standard laboratory diagnostics.

## 1 Introduction

The analysis of cerebrospinal fluid (CSF) is essential for the diagnosis of various neurological diseases. CSF findings like the color of the fluid or the CSF pressure are available immediately, while other parameters like cell count, erythrocytes in CSF, lactate, and glucose concentration are within hours, and some findings like an intrathecal immunoglobulin synthesis or CSF culture are usually not on the same day (1, 2). In time-critical diseases such as subarachnoid hemorrhage (SAH) or central nervous system infections, rapid diagnostic assessment is mandatory for an appropriate therapy (3–6). In SAH, a diagnostic and consequently therapeutic delay is associated with increased morbidity and long-term complications (4, 5). Also, in bacterial meningitis, delayed initiation of antibiotic therapy was linked to increased mortality (3, 6). Even in fully equipped hospitals, essential basic CSF parameters like cell count or lactate concentration for emergency treatment are not available instantly (7, 8). CSF samples need to be transferred to laboratories, in resource-limited surroundings even to external laboratories, resulting in an additional delay (7). Moreover, in some countries, comprehensive laboratory testing is often unavailable, complicating the diagnostic process (9). Therefore, a bedside screening test capable of detecting blood or markers of infection at the time of sampling may be particularly relevant in emergency departments, rural hospitals, and resource-limited settings where immediate access to specialized CSF laboratory diagnostics is unavailable (10, 11). In these settings, a simple bedside screening tool could potentially facilitate early identification of patients requiring urgent antimicrobial treatment or transfer to specialized care.

In urinary diagnostics, urine test strips (UTS) are widely used for rapid bedside assessment. UTS provide semi-quantitative estimates of leukocytes, erythrocytes, total protein, and glucose. These parameters are also part of CSF analysis. The rapid and simple testing procedure of UTS enables a fast-track diagnosis and therapeutic procedure like the start of an antibiotic therapy in case of an infection (12, 13).

Previous studies evaluated the use of UTS to detect blood in CSF and could show good correlation between UTS and laboratory results (14–16). Furthermore, other studies examining the performance to diagnose bacterial meningitis with UTS reported high sensitivity and specificity to detect CSF pleocytosis and elevated CSF protein levels (7, 8, 17–23). In contrast, Molyneux et al. reported a sensitivity of only 33% and a specificity of 83% for the diagnosis of bacterial meningitis using UTS (24). Therefore, the utility of UTS as a method for rapid CSF diagnostic is inconsistently between different studies, which are limited by using UTS from only one manufacturer (7, 8, 17–24).

The aim of the present study was therefore to further evaluate the diagnostic performance of UTS for CSF diagnostics. This was assessed employing a prospective real-world study involving comparison of clinically relevant parameters of UTS with laboratory measurements as reference and conducting a direct head-to-head comparison of five different UTS manufacturers using the same samples. Furthermore, we investigated the generalizability of existing data from selective meningitis cohorts to non-selective clinical practice, drawing on a broad spectrum of neurological disorders in our cohort. Additionally, we assessed the clinical diagnostic accuracy of UTS for differentiating infectious from non-infectious diseases, including separate analyses of bacterial and viral infections.

## 2 Methods

### 2.1 Patients

In this prospective study, consecutive CSF leftover samples from the laboratory for CSF diagnostics and neurochemistry at Hannover Medical School obtained in the time from 2019–2024 were used for UTS testing. CSF samples were obtained from patients evaluated at the Department of Neurology of Hannover Medical School, including the emergency department and neurological inpatient units. UTS testing was performed on a small fraction of the CSF samples only when the amount of CSF was more than required and only before routine laboratory CSF analysis was initiated. Clinical information and final diagnoses were obtained retrospectively from the medical records after completion of the standard diagnostic work-up. The investigator performing and interpreting the UTS results was blinded to both the suspected and final clinical diagnosis.

The diagnoses after standard CSF analysis compromised the following groups:

- Viral and bacterial infections (e.g., herpes simplex encephalitis or neuroborreliosis)
- Non-inflammatory neurological diseases [degenerative, peripheral, idiopathic, vascular neurological disorders (e.g., idiopathic intracranial hypertension, dementia, or polyneuropathy)]
- Autoimmune and paraneoplastic neurological disorders (e.g., multiple sclerosis (MS) or central nervous system (CNS) lymphoma)
- Non-neurological disorders (e.g., psychiatric disorders)

Patients were diagnosed in accordance with currently available guidelines by experienced, board-certified neurologists. In particular, diagnosis of neuroinfectious disease was made in accordance with the criteria published by Venkatesan et al. (25) and diagnosis of MS according to the 2017-revised McDonald criteria (26).

### 2.2 Laboratory analyses

CSF diagnostics were performed according to international standards and guidelines at the laboratory for CSF diagnostics and neurochemistry at Hannover Medical School (27–29). CSF cell counts were determined manually using a Fuchs-Rosenthal counting chamber. Total CSF protein was measured in centrifuged samples employing the Bradford dye-binding method. Albumin and immunoglobulin classes (IgG, IgA, and IgM) were quantified in paired serum and CSF samples with kinetic nephelometry (IMMAGE system, Beckman Coulter, Brea, CA, United States). Intrathecal production of immunoglobulins was calculated based on Reiber’s revised hyperbolic model (30, 31). Detection of CSF-restricted oligoclonal bands was performed by isoelectric focusing on polyacrylamide gels followed by silver staining (30, 31). Elevated values were defined as leukocytes > 4 cells/μL, erythrocytes > 0 cells/μl, protein > 500 mg/L, lactate > 2.1 mmol/L in patients < 50 years and > 2.6 mmol/L in patients ≥ 50 years, and QAlbumin > [(4 + age)/15]× 10^−3^.

When an infectious disease of the CNS was suspected, further CSF diagnostics including determination of intrathecally produced antibodies against varicella zoster (VZV), herpes simplex virus (HSV), influenza virus, Epstein–Barr virus (EBV), tick-borne encephalitis virus, and cytomegalovirus (CMV) and polymerase chain reactions analyses for these viruses could be performed according to the suspected pathogen. The Antibody-specific index (AI) was calculated using the following formula: (CSF virus-IgG/serum virus-IgG)/(CSF IgG total/serum IgG total) (32, 33). Detection of *Borrelia burgdorferi* as well as other bacterial pathogens in CSF and serum was conducted using commercial ELISA kits.

### 2.3 Urine test strips

UTS from five different manufacturers were used: Analyticon (Combi Screen 10SL Plus), Hitado (NobiStrip U10), Siemens (Multistix 10SG), Roche (Combur 10 UX), and Macherey-Nagel (Medi-Test Combi 10 SGL). Each strip contains 10 parameters including: protein, blood/erythrocytes, leukocytes, nitrite, glucose, ketone, pH, specific gravity, bilirubin, urobilinogen. The CSF parameters leukocytes, erythrocytes, protein, and glucose were tested with CSF samples.

As the semiquantitative segmentation differed between manufacturers, UTS results were harmonized into common categories to enable comparison across manufacturers. These categories were based on the ranges provided by the respective manufacturers and were not intended to represent clinically established CSF reference ranges:

- Erythrocytes (/μL): negative (0), slightly increased (1–80), strongly increased (> 80)
- Glucose (mg/dL): negative (0), low (1–100), moderate (101–500), high (> 500)
- Protein (mg/L): negative (< 500), slightly increased (500–3,000), strongly increased (> 3,000)
- Leukocytes (/μL): negative (0), low (1-25), moderate (26–75), high (> 75)

For context, the clinically established reference ranges for CSF were <5 cells/μL for leukocytes, <5 cells/μL for erythrocytes, <500 mg/L for total protein, and a CSF/serum glucose ratio >0.5 (1). These clinical reference ranges were distinguished from the manufacturer-defined UTS thresholds and from the study-specific thresholds used in the clinical diagnostic analysis.

For the first analysis assessing the analytical/parameter accuracy, laboratory results were dichotomized using the manufacturer-defined thresholds separating negative from positive UTS results. These thresholds were applied to the corresponding laboratory measurements to enable a direct comparison between the UTS and the laboratory reference method. According to the manufacturers’ package inserts, leukocyte counts >4 cells/μL and erythrocyte counts >5 cells/μL correspond to positive UTS results. For protein, a cut-off of 500 mg/L was applied to the laboratory measurements because this value corresponds both to the threshold separating negative from positive results in the strip-derived categorization and to the clinically established upper reference limit for CSF protein (1). For this analysis, UTS glucose results were not formally compared with laboratory CSF glucose measurements because CSF glucose measurements were available for only a limited number of samples.

For the secondary analysis assessing the clinical diagnostic accuracy, leukocytes, total protein, and glucose were analyzed. Based on the available semiquantitative UTS segmentation, study-specific thresholds for a positive result were defined as follows: leukocytes >0 cells/μL, protein >500 mg/L, and glucose <100 mg/dL. For leukocytes, the threshold of >0 cells/μL was used as the study-specific UTS threshold because the clinically relevant threshold of >4 cells/μL is not represented by the semiquantitative UTS scale. For glucose, the clinically established reference is based on the CSF/serum glucose ratio rather than on an absolute CSF glucose concentration. As the UTS provide absolute glucose categories, the clinical reference threshold could not be directly transferred to the UTS scale. Therefore, a study-specific threshold of <100 mg/dL was selected as the nearest available UTS category corresponding to a low CSF glucose concentration.

CSF samples were analyzed immediately after collection. Prior to testing, samples were not centrifuged. A drop of CSF (approx. 30 μL) was applied to each test field of the reagent strips. After the instructed time, the results were evaluated visually according to the manufacturer’s color scale. UTS testing was performed before results of the reference laboratory analyses were available, and the investigator performing and interpreting the UTS was blinded to the suspected and final clinical diagnosis. Conversely, UTS results were not available to the laboratory personnel performing the reference standard analyses or to the treating neurologists and were not considered when establishing the final clinical diagnosis.

### 2.4 Statistical analyses

Descriptive statistics were calculated for all variables. Continuous variables are presented as median and interquartile range (IQR) or mean ± standard deviation (SD), as appropriate.

To evaluate the analytical/parameter accuracy of UTS, UTS results were compared with the corresponding laboratory CSF measurements, which served as the reference standard. Spearman rank correlation was performed for erythrocytes, leukocytes, and protein. Subsequently, the number of results per category and parameter was counted and compared using Chi-square test and Fisher–Freeman–Halton test. Effect sizes were quantified using Cramer’s V. Diagnostic performance of the reagent strips was evaluated by comparing strip and laboratory categories using Chi-square test, as described above.

Sensitivity, specificity, positive predictive value (PPV), and negative predictive value (NPV), including 95% confidence intervals (95% CI), were calculated using Fisher’s exact test and Receiver operating curves (ROC) based on dichotomized (positive vs. negative) results with the laboratory measurements as the reference standard. ROC curve analyses were additionally performed using the harmonized ordinal UTS categories as the test variable and dichotomized laboratory measurements as the reference standard.

In a separate secondary analysis, UTS was assessed by evaluating their ability to differentiate infectious from non-infectious CNS disease using the parameters leukocytes, protein, and glucose. The final clinical diagnosis served as the reference standard. Each UTS parameter was first analyzed individually using Fisher’s exact test. Subsequently, combinations of parameters were evaluated to determine which cut-off value (≥1, ≥2, ≥3 positive parameters) yielded the highest sensitivity and specificity using Fisher’s exact test.

In a subgroup analysis, the same approach was used to evaluate the ability of UTS to differentiate bacterial infections from non-infectious diseases and viral infections from non-infectious diseases using leukocytes, protein, and glucose.

All statistical analyses were conducted using GraphPad Prism software (version 8.4.3) and IBM SPSS statistics (version 30.0.0). A two-sided *p*-value < 0.05 was considered statistically significant. For multiple comparisons, Bonferroni correction was applied where appropriate.

## 3 Results

### 3.1 Patients and CSF routine parameters

In total, CSF samples from 285 patients were analyzed. The median age was 55 years (IQR: 35–65 years) and 154/285 (54%) were female. The diagnostic spectrum comprised viral (27%) and bacterial infections (13%), non-inflammatory neurological diseases (degenerative, peripheral, idiopathic and vascular neurological disorders (21%)), autoimmune, para-, and neoplastic neurological conditions (27%), as well as non-neurological disorders (12%). Patients with neuroinfectious diseases were divided into the groups of viral and bacterial infections according to basic CSF parameters. Causing pathogens were detected in 48% of the patients in the viral infection group and in 86% of patients in the bacterial infection group. Pathogens were EBV, VZV, HSV, influenza virus, tick-borne encephalitis virus, CMV, *borrelia burgdorferi*, pneumococci, cryptococci, *staphylococcus aureus*, *staphylococcus hominis*, staphylococcus pyogenes, toxoplasma gondii, and *treponema pallidum*. Among these patients with infectious CNS diseases, 16.8% had relevant immunosuppression or immune dysfunction, including patients with transplantation, HIV infection, PML, or immunomodulatory treatment for MS.

Table 1 shows an overview of the total cohort and the diagnostic groups and their laboratory CSF findings.

**Table 1:** Demographic and cerebrospinal fluid routine parameters across the diagnostic groups.

Variable	All patients (*n* = 285)	Viral infection (*n* = 77)	Bacterial infection (*n* = 36)	Non-inflammatory neurological diseases* (*n* = 61)	Auto-immune or Paraneo-plastic neurological diseases (*n* = 77)	Non-neuro-logical diseases (*n* = 34)
Females, *n* (%)	154/285, (54)	36/77 (46.8)	17/36 (47.2)	36/61 (59)	47/77 (61)	18/34 (52.9)
Age [years], median (IQR)	55 (35–65)	52 (34–64)	58 (48.25–68.5)	61 (45–74)	53 (35–62)	38.5 (32–56.25)
CSF routine parameters						
CSF leukocytes [/μl], median (IQR)	10 (1.3–42.5)	35 (10.3–76)	127 (31.98–1,696)	1 (0.3–2.85)	16 (5.85–36.35)	1 (0.7–2)
CSF pleocytosis, *n* (%)	183/285 (64.2)	71/77 (92.2)	35/36 (97.2)	11/61 (18)	63/77 (81.8)	2/34 (5.9)
CSF erythrocytes [/μl], median (IQR)	2 (0.3–12)	4 (0.7–21.8)	4 (0.7–80)	1 (0.15–44.85)	1 (0.3–5)	0 (0–3.33)
CSF total protein [mg/L], median (IQR)	511 (394–722)	564.5 (418.5–867.8)	1,184 (594.5–3,778)	468 (370–555)	522 (403.5–726)	377 (331–445)
CSF total protein elevated, *n* (%)	151/279 (54.1)	47/74 (63.5)	28/34 (82.4)	25/60 (41.7)	46/77 (59.7)	5/34 (14.7)
CSF lactate [mmol/l], median (IQR)	1.75 (1.56–2.47)	1.89 (1.63–2.63)	2.76 (2.05–7.42)	1.56 (1.45–1.76)	1.98 (1.59–2.78)	1.58 (1.44–1.73)
CSF lactate elevated, *n* (%)	69/265 (26)	22/71 (31)	21/34 (61.8)	1/57 (1.8)	23/73 (31.5)	2/30 (6.7)
Albumin quotient, median (IQR)	7.01 (4.71–11.86)	7.61 (4.73–13.97)	20.35 (7.51–75.38)	6.4 (4.39–8.84)	7.58 (5.05–12.95)	4.82 (4.05–6.13)
Albumin quotient elevated, *n* (%)	130/280 (46.4)	41/75 (54.7)	28/34 (82.4)	17/60 (28.3)	39/77 (50.7)	5/34 (14.7)

CSF, cerebrospinal fluid; IQR, interquartile range; * = compromising degenerative, peripheral, vascular, idiopathic neurological disorders.

### 3.2 Analytical accuracy: comparison of urine test stripes and laboratory results

The analytical accuracy of the UTS was assessed by comparing the UTS results to the corresponding laboratory CSF measurements. Strong positive correlations were observed between UTS results of all manufacturers and laboratory measurements for erythrocytes (Spearman’s *ρ* = 0.74–0.84, all *p* < 0.001), protein (*ρ* = 0.61–0.69, all *p* < 0.001), and a moderate positive correlation for leukocytes (*ρ* = 0.4–0.41, all *p* < 0.001) (Figure 1).

**Figure 1:**
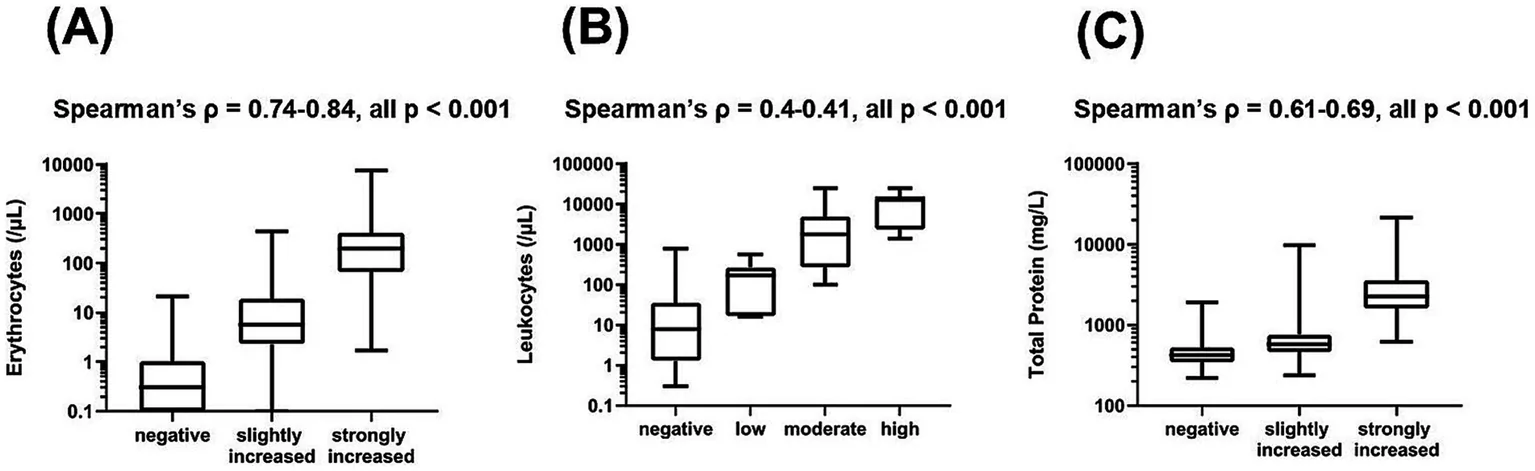
Distribution of laboratory values across UTS categories, pooled across from all manufacturers. Test strip categories are plotted against corresponding laboratory measurements for **(A)** erythrocyte count, **(B)** leukocyte count, and **(C)** total protein in CSF. Spearman’s rank correlation coefficients (*ρ*) and corresponding *p*-values are indicated in each panel.

#### 3.2.1 Detection of erythrocytes in CSF: high sensitivity and moderate specificity

UTS and manual erythrocyte detection via microscopy was significantly consistent for Medi-Test Combi 10 SGL (Macherey-Nagel) and NobiStrip U10 (Hitado) (Figure 1). Combi Screen 10SL Plus (Analyticon), Multistix 10SG (Siemens), and Combur 10 UX (Roche) differed but not statistically significant. All manufacturers demonstrated high sensitivity (85.7–94.9%) and high NPV (91.7–96.9%) with highest values yielded for Medi-Test Combi 10 SGL (Macherey-Nagel) as shown in Table 2. Specificity ranged from 79.6 to 83.3% and PPV ranged from 68.7 to 75% across manufacturers. ROC curve analysis for the detection of erythrocytes across pooled manufacturer demonstrated high diagnostic accuracy with an AUC of 0.87 (Figure 2). ROC analysis based on the harmonized UTS categories yielded an AUC of 0.92 for the detection of laboratory-defined erythrocyte positivity. At the first positive UTS category (>0/μL), sensitivity was 92.7% and specificity was 80.8%. Increasing the threshold to >80/μL increased specificity to 99.8%, while sensitivity decreased to 50.6%. Detailed results are shown in Supplementary Figure S1.

**Table 2:** Diagnostic performance of UTS for detection of CSF blood contamination (> 5 erythrocytes/μL) using laboratory CSF erythrocyte count as the reference standard.

Variable	Combi screen 10SL plus (Analyticon)	NobiStrip U10 (Hitado)	Multistix 10SG (Siemens)	Combur 10 UX (Roche)	Medi-test combi 10 SGL (Macherey-Nagel)
Sensitivity, % (95% CI)	93.9 (87.3–97.2)	85.7 (77.4–91.3)	93.9 (87.3–97.2)	94.9 (88.6–97.8)	94.9 (88.6–97.8)
Specificity, % (95% CI)	79.6 (73.2–84.7)	83.3 (77.3–88)	77.4 (70.9–82.8)	80.1 (73.8–85.2)	83.3 (77.3–88)
PPV, % (95% CI)	70.8 (62.4–77.9)	73 (64.3–80.3)	68.7 (60.4–75.9)	71.5 (63.3–78.6)	75 (66.7–81.8)
NPV, % (95% CI)	96.1 (91.8–98.2)	91.7 (86.6–95)	96 (91.6–98.2)	96.8 (92.6–98.6)	96.9 (92.9–98.7)

PPV, Positive predictive value; NPV, Negative predictive value.

**Figure 2:**
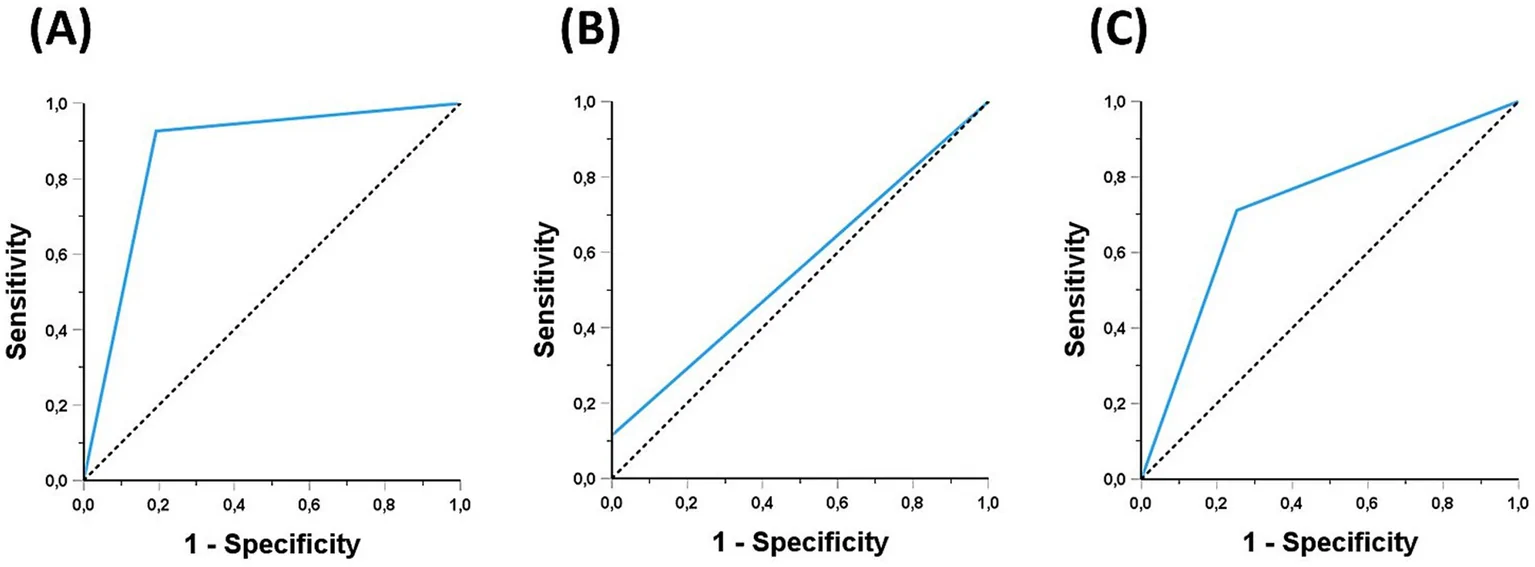
ROC analysis of dichotomized UTS results (positive vs. negative). Data were pooled across from all manufacturers with laboratory measurements as the reference standard, for the detection of **(A)** Erythrocytes (>5 cells/μL), **(B)** Pleocytosis (≥5 cells/μL), and **(C)** Elevated total protein (≥500 mg/L) in CSF. Blue line: ROC curve; dashed line: reference line indicating an AUC of 0.5.

#### 3.2.2 Detection of pleocytosis: low sensitivity and high specificity

The detection of leukocytic pleocytosis in CSF differed significantly from the cell count found by the standard method across the different UTS. For CSF pleocytosis (> 4 leukocytes/μL in laboratory testing), no false-positive strip results were observed across all manufacturers, resulting in a specificity and PPV of 100%. Sensitivity was low ranging between 11 and 11.6%, and the NPV was ranging between 39.4 and 39.5%. Results are summarized in Table 3 and Figure 1. ROC curve analysis for the detection of pleocytosis across pooled manufacturer demonstrated low diagnostic accuracy with an AUC of 0.56 (Figure 2). ROC analysis based on the harmonized UTS categories yielded an AUC of 0.56. At the first positive UTS category (1–25/μL), sensitivity was 11.4% and specificity was 100%. Increasing the threshold to 26–75/μL and >76/μL further reduced sensitivity to 7.9 and 3.8%, respectively, while specificity remained at 100%. At the highest threshold, sensitivity decreased to 0%, with specificity remaining at 100% (Supplementary Figure S1).

**Table 3:** Diagnostic performance of UTS for detection of CSF pleocytosis (> 4 leukocytes/μL) compared to detection of pleocytosis in the laboratory.

Variable	Combi screen 10SL plus (Analyticon)	NobiStrip U10 (Hitado)	Multistix 10SG (Siemens)	Combur 10 UX (Roche)	Medi-test combi 10 SGL (Macherey-Nagel)
Sensitivity, % (95% CI)	11.6 (7.7–17.1)	11.05 (7.3–16.5)	11.6 (7.7–17.1)	11.6 (7.7–17.1)	11.6 (7.7–17.1)
Specificity, % (95% CI)	100 (96.4–100)	100 (96.5–100)	100 (96.4–100)	100 (96.4–100)	100 (96.4–100)

PPV, Positive predictive value; NPV, Negative predictive value.

#### 3.2.3 Detection of elevated CSF protein: manufacturer depending low to high sensitivity and specificity

Protein strip results differed significantly from laboratory measurements across all manufacturers. Combi Screen 10SL Plus (Analyticon) and Multistix 10SG (Siemens) more frequently classified samples as “slightly increased” or “strongly increased” compared with laboratory findings. In contrast, NobiStrip U10 (Hitado), Combur 10 UX (Roche), and Medi-Test Combi 10 SGL (Macherey-Nagel) more often yielded negative results and less frequently classified samples as elevated. In the dichotomized analysis comparing positive to negative results, Combi Screen 10SL Plus (Analyticon) and Multistix 10SG (Siemens) showed higher sensitivity (88 and 90.8%) and NPV (82.4 and 82%), whereas NobiStrip U10 (Hitado), Combur 10 UX (Roche), and Medi-Test Combi 10 SGL (Macherey-Nagel) demonstrated higher specificity (80.5%, 84.4%, and 93%) and PPV (75.1%, 82.8%, and 89.5%). Detailed results are shown in Table 4 and Figure 1. ROC curve analysis for the detection of elevated protein values across pooled manufacturer demonstrated moderate diagnostic accuracy, with an AUC of 0.73 (Figure 2), yielding moderate sensitivity (49.5%) and specificity (54.1%). ROC analysis based on the harmonized UTS categories yielded an AUC of 0.75. At the first positive UTS category (500–3,000 mg/L), sensitivity was 71.1% and specificity was 74.7%. A threshold corresponding to the strongly increased protein category (>3,000 mg/L) increased specificity to 100%, but reduced sensitivity to 18.5% (Supplementary Figure S1).

**Table 4:** Diagnostic performance of UTS for detection of elevated protein values in CSF (> 500 mg/L) using laboratory CSF protein measurement as the reference standard.

Variable	Combi screen 10SL plus (Analyticon)	NobiStrip U10 (Hitado)	Multistix 10SG (Siemens)	Combur 10 UX (Roche)	Medi-test combi 10 SGL (Macherey-Nagel)
Sensitivity, % (95% CI)	88.1 (81.9–92.3)	62.3 (54.3–69.6)	90.7 (85–94.4)	63.6 (55.7–70.8)	51 (43.1–58.8)
Specificity, % (95% CI)	65.6 (57–73.3)	80.5 (72.8–86.4)	50 (41.5–58.5)	84.4 (77.1–89.7)	93 (87.2–96.3)

### 3.3 Detailed inter-manufacturer comparison of urine test stripes

The detection of erythrocytes was consistent in the categories “negative,” “slightly increased,” and “strongly increased” among all UTS.

Also, leukocyte detection did not differ significantly between the different manufactures.

The detection of elevated protein in the category “strongly increased” did not differ significantly between manufacturers. In contrast, both the “negative” and “slightly increased” categories showed significant inter-manufacturer differences (both *p* < 0.001), with moderate effect sizes (Cramer’s V = 0.298 and 0.317, respectively). Higher detection rates in the “normal” category were observed with Medi-Test Combi 10 SGL (Macherey-Nagel), whereas Multistix 10SG (Siemens) most frequently classified samples as “slightly increased.” More detailed results are provided in Supplementary Table S1.

A significant difference was observed only in the “low” category for glucose (*p* < 0.001), where Combur 10 UX (Roche) showed higher detection rates than the other manufacturers with a small effect size (Cramer’s V = 0.135).

### 3.4 Clinical diagnostic accuracy: discrimination of infectious and non-infectious samples using urine test strips

To assess the utility of UTS to discriminate infectious from non-infectious samples, sensitivity, specificity, and PPV and NPV were calculated in a univariate analysis for the parameters elevated leukocytes, glucose, protein and in a multivariate analysis for the combination of all three.

Pathological glucose and leukocyte levels demonstrated high specificity and PPV across all manufacturers (overall specificity: leukocytes 97.7%, glucose 99.4–100%; overall PPV: leukocytes 80%, glucose 85.7–100%) for detecting an infectious disease of the CNS. In contrast, elevated protein was of lower specificity and PPV (overall specificity 29.7–75.6%; overall PPV 41–53.3%). Sensitivity and NPV were low to moderate for all parameters (overall sensitivity: leukocytes 14.2–15%, glucose 4.4–5.3%, protein 42.5–74.3%; overall NPV: leukocytes 63.3–63.4%, glucose 61.4–61.7%, protein 63–66.7%). Detailed results are provided in Supplementary Tables S2, S3.

Inter-manufacturer differences were not significant for elevated leukocytes and glucose. For elevated protein, Medi-Test Combi 10 SGL (Macherey-Nagel) demonstrated the highest specificity, PPV, and NPV (75.6%, 53.3%, and 66.7%, respectively), while Multistix 10SG (Siemens) showed the highest sensitivity (74.3%).

With a cut-off of at least 2/3 pathological parameters (elevated leukocytes, elevated protein or reduced glucose) specificity from 97.7 to 98.3% and PPV from 80 to 85% were found as shown in Table 5. Results of the cut-offs 1/3 and 3/3 pathological parameters can be found in the Supplementary Tables 4, 5.

**Table 5:** Diagnostic performance of UTS for detection of infectious versus non-infectious samples if ≥ 2/3 parameters (elevated leukocytes and protein and reduced glucose) were tested positive.

Variable	Combi screen 10SL plus (Analyticon)	NobiStrip U10 (Hitado)	Multistix 10SG (Siemens)	Combur 10 UX (Roche)	Medi-test combi 10 SGL (Macherey-Nagel)
Sensitivity, % (95% CI)	14.2 (8.9–21.8)	13.3 (8.2–20.8)	15 (9.6–22.8)	14.2 (8.9–21.8)	14.2 (8.9–21.8)
Specificity, % (95% CI)	97.7 (94.1–99.1)	98.3 (95–99.5)	98.3 (95–99.5)	98.3 (95–99.5)	98.3 (95–99.5)
PPV, % (95% CI)	80 (58.4–91.9)	83.3 (60.8–94.2)	85 (64–94.8)	84.2 (62.4–94.5)	84.2 (62.4–94.5)
NPV, % (95% CI)	63.4 (57.4–69)	63.3 (57.4–68.9)	63.8 (57.8–69.3)	63.5 (57.6–69.1)	63.5 (57.6–69.1)

PPV, Positive predictive value; NPV; Negative predictive value.

In a subgroup analysis, we assessed the clinical diagnostic accuracy of UTS for differentiating bacterial infections from non-infectious diseases and viral infections from non-infectious diseases. For bacterial infections, pathological leukocyte and glucose levels showed high specificity (leukocytes 97.67%; glucose 99.42–100%), PPV (leukocytes 77.78%; glucose 83.33–100%) and NPV (leukocytes 88.42%; glucose 84.65–84.73%), whereas sensitivity was low (leukocytes 38.89%; glucose 13.89%). When combining all three parameters (leukocytes, protein, glucose), using a cut-off of ≥ 2 of 3 pathological parameters resulted in high values for specificity (97.67–98.26%), PPV (77.78–82.35%), and NPV (88.42–88.48%), while sensitivity remained low (38.89%). Detailed results are presented in Supplementary Table S6.

For viral infections, leukocyte results alone showed high specificity (97.67%), moderate PPV (33.33–42.86%) and NPV (69.14–69.42%), whereas sensitivity was low (2.6–3.9%). Glucose showed high specificity (99.42–100%) and moderate NPV (69.08%–69.35%), while PPV varied between manufacturers (Combi Screen 10SL Plus (Analyticon) 50%; Combur 10 UX (Roche) 100%). For NobiStrip U10 (Hitado), Multistix 10SG (Siemens), and Medi-Test Combi 10 SGL (Macherey-Nagel) the PPV could not be calculated because no positive UTS results were observed in either the viral or non-infectious group. When applying a cut-off of ≥ 2/3 pathological parameters, specificity remained high (97.67–98.26%), whereas NPV was moderate (68.98–69.55%) and sensitivity and PPV were low (overall sensitivity 1.3–3.9%; overall PPV 25–50%) (Supplementary Table S1).

## 4 Discussion

In this study, we evaluated the analytical and clinical diagnostic accuracy, and inter-manufacturer comparability of UTS for CSF analysis in time-critical clinical settings.

The first main finding of this study is that UTS results were positively correlated with laboratory measurements across all tested parameters and manufacturers. For detecting erythrocytes, UTS showed high sensitivity and moderate specificity in CSF compared to the laboratory findings, detected in a specialized CSF laboratory. In contrast, UTS had low sensitivity but high specificity for detecting pleocytosis compared with the laboratory results. For elevated protein, the diagnostic performance varied between the manufacturers, some showing higher sensitivity and others higher specificity. Erythrocyte detection showed robust performance with high sensitivity and NPV, supporting the use of UTS to reliably exclude blood contamination and highlighting their usefulness in time-critical and resource-limited settings, consistent with previous studies (7–9, 12, 15). Clinically, this may simplify diagnostic workflows: a negative UTS result for erythrocytes could help rule out blood in CSF, potentially reducing the need for additional diagnostic procedures and therefore saving time and resources. In contrast, specificity and PPV for detecting pleocytosis were consistently high due to the absence of false-positive results. The low sensitivity is likely explained by the insufficient resolution of semi-quantitative strip categories of UTS, which led to systematic underestimation of low leukocyte counts, especially around the diagnostic threshold of 4 cells/μL (1). Thus, a positive leukocyte result on UTS may provide useful evidence for pleocytosis, whereas a negative result cannot reliably exclude it. This limitation was also been described in broader, unselected cohorts (24). For protein analysis, test performance for detecting elevated total CSF protein (cut-off 500 mg/L) demonstrated strengths depending on the manufacturer, with either sensitivity or specificity predominating (1). This variability was reported previously and was likely attributable to differences in strip calibration and cut-offs rather than biological variability (7, 8, 17). Overall, these findings together with the moderate diagnostic accuracy shown by the ROC analysis highlight a mismatch between urine-based semi-quantitative scales and clinically relevant CSF thresholds particularly for detecting pleocytosis. ROC analysis based on the harmonized UTS categories further supported this finding, as increasing the threshold to higher UTS categories did not improve diagnostic discrimination but resulted in a marked loss of sensitivity.

The second main finding is that the clinical diagnostic accuracy of UTS did not significantly differ among the different manufactures. Evaluation of protein are most likely due to categorization and cut-off definitions than in different measurements. This observation is of relevance for clinical practice, as previous studies almost exclusively evaluated single manufacturers, most commonly Combur 10 UX (Roche) or Multistix 10SG (Siemens), and direct head-to-head comparisons were lacking (18, 34). Our results therefore suggest that diagnostic performance of UTS in CSF is largely independent of the manufacturer and support their general applicability across different brands.

Lastly, we tested the ability of UTS to differentiate infectious from non-infectious conditions. Elevated leukocytes and reduced glucose demonstrated high specificity and PPV, particularly when multiple parameters were positive. Using a cut-off of ≥ 2 out of 3 positive parameters (leukocytes, glucose, and protein), specificity and PPV were consistently high among manufacturers. This is in line with previous studies in bacterial meningitis reporting high specificity for leukocyte esterase and glucose-based strip parameters (21–23). However, sensitivity and NPV remained low, limiting the ability of UTS to reliably exclude infection. This discrepancy compared to earlier reports with higher sensitivities is likely explained by differences in study populations, as previous studies often focused on selected meningitis cohorts with high-grade pleocytosis (7, 8, 17–19), whereas our cohort reflects a broader clinical spectrum. This interpretation is supported by our subgroup analysis, which demonstrated higher diagnostic accuracy for differentiating bacterial infections from non-infectious diseases than for differentiating viral infections from non-infectious diseases. This may be explained by the typically more pronounced CSF abnormalities in bacterial CNS infections, including high-grade pleocytosis, often exceeding 1,000 cells/μL, and elevated protein concentrations with more than 1,000 mg/L (1). In contrast, viral CNS infections often present with less pronounced or more variable CSF abnormalities, limiting the ability of UTS to detect these cases (1). Consequently, given their high specificity and PPV, UTS should be interpreted as a rule-in rather than a rule-out tool for CNS infection. These findings may be particularly relevant in resource-limited settings or clinical situations where immediate access to specialized CSF laboratory diagnostics is unavailable. A positive result, particularly when different parameters are abnormal, may increase the suspicion of an infectious CNS process and support early decision-making and prioritization of urgent diagnostic and therapeutic measures. Conversely, a negative UTS result cannot reliably exclude CNS infection and should neither replace standard CSF laboratory diagnostics nor delay further diagnostic or therapeutic measures when infection is clinically suspected.

From a methodological perspective, the observed limitations underscore the need for CSF-specific test systems. Current UTS are not calibrated for clinically relevant CSF ranges, particularly low leukocyte counts and higher protein concentrations. Adaptation of cut-offs and improved resolution in diagnostically critical ranges would likely enhance their performance substantially. Especially with regard to CSF glucose, UTS have limited clinical applicability, as CSF glucose is typically interpreted as a CSF/serum glucose ratio rather than as an absolute CSF glucose level. In addition, predictive values are strongly dependent on disease prevalence. The relatively high prevalence of pleocytosis and heterogeneous case mix in our cohort likely contributed to the observed low NPV and wide confidence intervals. Therefore, diagnostic accuracy may differ in other clinical settings. In addition, information on clinically documented traumatic lumbar punctures was not systematically available. Therefore, erythrocytes detected in CSF could not be reliably differentiated according to their origin.

The limitations are further explained by the fact that UTS are designed for urine rather than CSF. Differences in physiological conditions, such as pH and protein composition, may affect the underlying colorimetric reactions and thereby reduce analytical accuracy. Consequently, UTS are better suited for binary clinical decisions (e.g., presence vs. absence of pathological findings) than for precise quantification.

In conclusion, UTS provide a rapid, low-cost bedside tool that can support early CSF assessment, particularly in emergency or resource-limited settings. Their strengths lie in reliably excluding blood in CSF. UTS can also identify patients with a high likelihood of CNS infection, particularly bacterial infections, when at least 2/3 parameters of leukocytes, protein and glucose are pathological. Due to limited sensitivity and systematic underestimation of low-grade pleocytosis, UTS cannot replace standard laboratory diagnostics and should be interpreted as a complementary, rule-in tool rather than a definitive test. Future developments of CSF-specific test strips with adapted cut-offs may substantially improve their clinical utility.

## Data Availability

The original contributions presented in the study are included in the article/Supplementary material, further inquiries can be directed to the corresponding author.

## Supplementary material

SUPPLEMENTARY FIGURE S1 ROC analysis based on the harmonized UTS categories pooled across from all manufacturers with laboratory measurements as the reference standard, for the detection of **(A)** erythrocytes (> 5 cells/µL), **(B)** pleocytosis (≥ 5 cells/µL), and **(C)** elevated total protein (≥ 500 mg/L) in CSF. Blue line: ROC curve; dashed line: reference line indicating an AUC of 0.5.
