# Supplementary material for: Diagnostic accuracy of urine test strips for CSF analysis: high sensitivity in detecting blood, low sensitivity but high specificity in detecting inflammation
Source: Front Neurol. 2026 Oct 26;17:1893760. doi: 10.3389/fneur.2026.1893760 (PMC13745362; doi:10.3389/fneur.2026.1893760)
Supplement: Supplementary file 1 [file Table_1.DOCX]

Supplementary Material

**Table 1.** Number of detected tests in each category for each parameter and manufacturer in comparison to the laboratory findings.

| Characteristic | Laboratory testing | Combi Screen 10SL Plus (Analyticon) | NobiStrip U10 (Hitado) | Multistix 10SG (Siemens) | Combur 10 UX (Roche) | Medi-Test Combi 10 SGL (Macherey-Nagel) |
| --- | --- | --- | --- | --- | --- | --- |
| Leukocytes, category negative (0 cells/µl), n | 104/285 | 264/ 285 | 265/ 285 | 264/ 285 | 264/ 285 | 264/ 285 |
| Leukocytes, category low (1-25 cells/µl), n | 75/285 | 9/285 | 6/285 | 5/285 | 5/285 | 7/285 |
| Leukocytes, category moderate (26-75 cells/µl), n | 56/285 | 7/285 | 7/285 | 7/285 | 9/285 | 7/285 |
| Leukocytes, category high (> 76 cells/µl), n | 50/285 | 5/285 | 7/285 | 9/285 | 7/285 | 7/285 |
|  |  | p < 0.001 | p < 0.001 | p < 0.001 | p < 0.001 | p < 0.001 |
| Protein category normal (< 500 mg/L), n | 128/278 | 104/285 | 162/285 | 80/285 | 165/285 | 195/285 |
| Protein category slightly increased (500-3000 mg/L), n | 138/278 | 147/285 | 102/285 | 181/285 | 83/285 | 58/285 |
| Protein category strongly increased (> 3000 mg/L), n | 12/278 | 34/285 | 21/285 | 24/285 | 37/285 | 32/285 |
|  |  | p = 0.001 | p = 0.003 | p < 0.001 | p < 0.001 | p < 0.001 |
| Erythrocytes category negative (0 cells/µl), n | 186/285 | 154/285 | 169/285 | 150/285 | 154/285 | 160/285 |
| Erythrocytes category slightly increased (1-80 cells/µl), n | 57/285 | 82/285 | 68/285 | 83/285 | 77/285 | 73/285 |
| Erythrocytes category strongly increased (>80 cells/µl), n | 41/285 | 48/285 | 47/285 | 51/285 | 53/285 | 51/285 |
|  |  | p = 0.018 | p = 0.33 | p = 0.008 | p = 0.023 | p = 0.082 |

**Table 2**. Diagnostic performance of urine test strips for discrimination of infectious versus non-infectious on the basis of detection of pleocytosis (> 4 cells/µL). PPV: positive predictive value. NPV: negative predictive value.

|  | Combi Screen 10SL Plus (Analyticon) | NobiStrip U10 (Hitado) | Multistix 10SG (Siemens) | Combur 10 UX (Roche) | Medi-Test Combi 10 SGL (Macherey-Nagel) |
| --- | --- | --- | --- | --- | --- |
| Sensitivity, %  (95% CI) | 15  (9.6-22.8) | 14.2  (8.9-21.8) | 15  (9.6-22.8) | 15  (9.6-22.8) | 15  (9.6-22.8) |
| Specificity, %  (95% CI) | 97.7  (94.2-99.1) | 97.7  (94.2-99.1) | 97.7  (94.2-99.1) | 97.7  (94.2-99.1) | 97.7  (94.2-99.1) |
| PPV, %  (95% CI) | 81  (60-92.3) | 80  (58.4-91.9) | 81  (60-92.3) | 81  (60-92.3) | 81  (60-92.3) |
| NPV, %  (95% CI) | 63.6  (57.7-69.2) | 63.4  (57.4-69) | 63.6  (57.7-69.2) | 63.6  (57.7-69.2) | 63.6  (57.7-69.2) |

**Table 3**. Diagnostic performance of urine test strips for discrimination of infectious versus non-infectious on the basis of decreased glucose concentration (< 100 mg/dL). PPV: positive predictive value. NPV: negative predictive value.

|  | Combi Screen 10SL Plus (Analyticon) | NobiStrip U10 (Hitado) | Multistix 10SG (Siemens) | Combur 10 UX (Roche) | Medi-Test Combi 10 SGL (Macherey-Nagel) |
| --- | --- | --- | --- | --- | --- |
| Sensitivity, %  (95% CI) | 5.3  (2.5-11.1) | 4.4  (1.9-9.9) | 4.4  (1.9-9.9) | 5.3  (2.5-11.1) | 4.4  (1.9-9.9) |
| Specificity, %  (95% CI) | 99.42  (93.4-98.8) | 100  (97.8-100) | 100  (97.8-100) | 100  (97.8-100) | 100  (97.8-100) |
| PPV, %  (95% CI) | 85.7  (48.7-99.3) | 100  (56.6-100) | 100  (56.6-100) | 100  (61-100) | 100  (56.6-100) |
| NPV, %  (95% CI) | 61.5  (55.7-67) | 61.4  (55.6-66.9) | 61.4  (55.6-66.9) | 61.4  (55.6-66.9) | 61.4  (55.6-66.9) |

**Table 4**. Diagnostic performance of UTS for detection of infectious versus non-infectious samples if ≥ 1/3 parameters (leukocytes, glucose and protein) were tested positive. PPV: positive predictive value. NPV: negative predictive value.

|  | Combi Screen 10SL Plus (Analyticon) | NobiStrip U10 (Hitado) | Multistix 10SG (Siemens) | Combur 10 UX (Roche) | Medi-Test Combi 10 SGL (Macherey-Nagel) |
| --- | --- | --- | --- | --- | --- |
| Sensitivity, %  (95% CI) | 70.8  (61.8-78.4) | 47.8  (38.8-56.9) | 74.3  (65.6-81.5) | 49.6  (40.5-58.6) | 43.4  (34.6-52.6) |
| Specificity, %  (95% CI) | 39.5  (32.5-47) | 58.7  (51.3-65.8) | 29.1  (22.8-36.3) | 61.1  (53.6-68) | 75  (68-80.9) |
| PPV, %  (95% CI) | 43.5  (36.5-50.7) | 43.2  (34.9-52) | 40.8  (34.3-47.6) | 45.6  (37-54.3) | 53.3  (43.1-63.1) |
| NPV, %  (95% CI) | 67.3  (57.7-75.7) | 63.1  (55.4-70.2) | 63.3  (52.3-73.1) | 64.8  (57.2-71.8) | 66.8  (59.9-73.1) |

**Table 5**. Diagnostic performance of UTS for detection of infectious versus non-infectious samples if > 3/3 parameters (leukocytes, glucose and protein) were tested positive. PPV: positive predictive value. NPV: negative predictive value.

|  | Combi Screen 10SL Plus (Analyticon) | NobiStrip U10 (Hitado) | Multistix 10SG (Siemens) | Combur 10 UX (Roche) | Medi-Test Combi 10 SGL (Macherey-Nagel) |
| --- | --- | --- | --- | --- | --- |
| Sensitivity, %  (95% CI) | 4.4  (1.9-9.9) | 4.4  (1.9-9.9) | 4.4  (1.9-9.9) | 4.4  (1.9-9.9) | 4.4  (1.9-9.9) |
| Specificity, %  (95% CI) | 100  (97.8-100) | 100  (97.8-100) | 100  (97.8-100) | 100  (97.8-100) | 100  (97.8-100) |
| PPV, %  (95% CI) | 100  (56.6-100) | 100  (56.6-100) | 100  (56.6-100) | 100  (56.6-100) | 100  (56.6-100) |
| NPV, %  (95% CI) | 61.4  (55.6-66.9) | 61.4  (55.6-66.9) | 61.4  (55.6-66.9) | 61.4  (55.6-66.9) | 61.4  (55.6-66.9) |

**Table 6**. Subgroup analysis: Diagnostic performance of UTS for detection of viral infections versus non-infectious samples and bacterial infections versus non-infectious samples on the basis of detection of pleocytosis (> 4 cells/µL), of decreased glucose concentration (< 100 mg/dL), or if ≥ 2/3 parameters (leukocytes, glucose and protein) were tested positive. PPV: positive predictive value. NPV: negative predictive value.

| Subgroup | Parameter |  | Combi Screen 10SL Plus (Analyticon) | NobiStrip U10 (Hitado) | Multistix 10SG (Siemens) | Combur 10 UX (Roche) | Medi-Test Combi 10 SGL (Macherey-Nagel) |
| --- | --- | --- | --- | --- | --- | --- | --- |
| Viral infection | Leukocytes | Sensitivity, %  (95% CI) | 3.9 (1.06-10.84) | 2.6 (0.46-8.99) | 3.9 (1.06-10.84) | 3.9 (1.06-10.84) | 3.9 (1.06-10.84) |
|  |  | Specificity, %  (95% CI) | 97.67  (94.17-99.09) | 97.67  (94.17-99.09) | 97.67  (94.17-99.09) | 97.67  (94.17-99.09) | 97.67  (94.17-99.09) |
|  |  | PPV, %  (95% CI) | 42.86 (15.82-74.95) | 33.33 (5.92-70) | 42.86 (15.82-74.95) | 42.86 (15.82-74.95) | 42.86 (15.82-74.95) |
|  |  | NPV, %  (95% CI) | 69.42 (63.35-74.89) | 69.14 (63.07-74.61) | 69.42 (63.35-74.89) | 69.42 (63.35-74.89) | 69.42 (63.35-74.89) |
|  | Glucose | Sensitivity, %  (95% CI) | 1.3  (0.07-7) | 0  (0-4.75) | 0  (0-4.75) | 1.3  (0.07-7) | 0  (0-4.75) |
|  |  | Specificity, %  (95% CI) | 99.42  (96.78-100) | 100  (97.82-100) | 100  (97.82-100) | 100  (97.82-100) | 100  (97.82-100) |
|  |  | PPV, %  (95% CI) | 50  (2.57-97.44) |  |  | 100  (5.13-100) |  |
|  |  | NPV, %  (95% CI) | 69.23 (63.22-74.66) | 69.08 (63.08-74.49) | 69.08 (63.08-74.49) | 69.35 (63.36-74.76) | 69.08 (63.08-74.49) |
|  | ≥ 2/3 pathological Parameters | Sensitivity, %  (95% CI) | 2.6 (0.46-8.99) | 1.3  (0.07-7) | 3.9 (1.06-10.84) | 2.6 (0.46-8.99) | 2.6 (0.46-8.99) |
|  |  | Specificity, %  (95% CI) | 97.67  (94.17-99.09) | 98.26  (95-99.52) | 98.26  (95-99.52) | 98.26  (95-99.52) | 98.26  (95-99.52) |
|  |  | PPV, %  (95% CI) | 33.33 (5.92-70) | 25  (1.28-69.94) | 50  (18.76-81.24) | 40  (7.11-76.93) | 40  (7.11-76.93) |
|  |  | NPV, %  (95% CI) | 69.14 (63.07-74.61) | 68.98 (62.93-74.44) | 69.55 (63.49-74.99) | 69.26 (63.21-74.72) | 69.26 (63.21-74.72) |
| Bacterial infection | Leukocytes | Sensitivity, %  (95% CI) | 38.89 (24.78-55.14) | 38.89 (24.78-55.14) | 38.89 (24.78-55.14) | 38.89 (24.78-55.14) | 38.89 (24.78-55.14) |
|  |  | Specificity, %  (95% CI) | 97.67  (94.17-99.09) | 97.67  (94.17-99.09) | 97.67  (94.17-99.09) | 97.67  (94.17-99.09) | 97.67  (94.17-99.09) |
|  |  | PPV, %  (95% CI) | 77.78 (54.79-91) | 77.78 (54.79-91) | 77.78 (54.79-91) | 77.78 (54.79-91) | 77.78 (54.79-91) |
|  |  | NPV, %  (95% CI) | 88.42 (83.09-92.23) | 88.42 (83.09-92.23) | 88.42 (83.09-92.23) | 88.42 (83.09-92.23) | 88.42 (83.09-92.23) |
|  | Glucose | Sensitivity, %  (95% CI) | 13.89 (6.08-28.66) | 13.89 (6.08-28.66) | 13.89 (6.08-28.66) | 13.89 (6.08-28.66) | 13.89 (6.08-28.66) |
|  |  | Specificity, %  (95% CI) | 99.42 (96.78-99.97) | 100 (97.82-100) | 100 (97.82-100) | 100 (97.82-100) | 100 (97.82-100) |
|  |  | PPV, %  (95% CI) | 83.33 (43.65-99.15) | 100 (56.55-100) | 100 (56.55-100) | 100 (56.55-100) | 100 (56.55-100) |
|  |  | NPV, %  (95% CI) | 84.65 (79.04-88.97) | 84.73 (79.14-89.03) | 84.73 (79.14-89.03) | 84.73 (79.14-89.03) | 84.73 (79.14-89.03) |
|  | ≥ 2/3 pathological Parameters | Sensitivity, %  (95% CI) | 38.89 (24.78-55.14) | 38.89 (24.78-55.14) | 38.89 (24.78-55.14) | 38.89 (24.78-55.14) | 38.89 (24.78-55.14) |
|  |  | Specificity, %  (95% CI) | 97.67  (94.17-99.09) | 98.26  (95-99.52) | 98.26  (95-99.52) | 98.26  (95-99.52) | 98.26  (95-99.52) |
|  |  | PPV, %  (95% CI) | 77.78 (54.79-91) | 82.35 (58.97-93.81) | 82.35 (58.97-93.81) | 82.35 (58.97-93.81) | 82.35 (58.97-93.81) |
|  |  | NPV, %  (95% CI) | 88.42 (83.09-92.23) | 88.48 (83.18-92.27) | 88.48 (83.18-92.27) | 88.48 (83.18-92.27) | 88.48 (83.18-92.27) |
